# Duodenal Histoplasmosis Presenting with Upper Gastrointestinal Bleeding in an AIDS Patient

**DOI:** 10.1155/2012/515872

**Published:** 2012-10-03

**Authors:** Michael A. Spinner, Heather N. Paulin, C. William Wester

**Affiliations:** ^1^Vanderbilt University School of Medicine, 215 Light Hall, Nashville, TN 37232-2582, USA; ^2^Division of Infectious Diseases, Vanderbilt University School of Medicine, A2200 MCN, 1161 21st Avenue South, Nashville, TN 37232-2582, USA; ^3^Vanderbilt Institute for Global Health (VIGH), 2525 West End Avenue, Suite 750, Nashville, TN 37203-1738, USA

## Abstract

Gastrointestinal histoplasmosis (GIH) is common in patients with disseminated disease but only rarely comes to clinical attention due to the lack of specific signs and symptoms. We report the unusual case of a 33-year-old Caucasian male with advanced AIDS who presented with upper GI bleeding from diffuse erosions throughout the duodenum. Biopsy of the lesions revealed small bowel mucosa with granulomatous inflammation and macrophages with small intracellular yeasts consistent with disseminated histoplasmosis. The patient demonstrated significant clinical improvement following a two-week course of liposomal amphotericin B. To our knowledge, this is the first case report of duodenal histoplasmosis leading to clinically significant bleeding, manifesting with worsening anemia and melanotic stools. Given our findings, we maintain that GIH should be considered on the differential diagnosis for GI bleeding in AIDS patients at risk, specifically those with advanced immunosuppression (i.e., CD4^+^ cell counts <100 cells/mm^3^) who reside in endemic areas (Ohio or Mississippi river valleys) and/or have a prior history of histoplasmosis. For diagnostic evaluation, we recommend checking a urine *Histoplasma* quantitative antigen EIA as well as upper and/or lower endoscopy with biopsy. We recommend treatment with a two-week course of liposomal amphotericin B followed by indefinite itraconazole.

## 1. Introduction

Histoplasmosis is the most common endemic fungal infection in the United States [1]. While infections are typically self-limited in immunocompetent individuals, patients with impaired T-cell immunity are susceptible to developing acute pulmonary infection and subsequent disseminated disease [2]. In the Ohio and Mississippi river valleys where the organism is endemic, disseminated histoplasmosis remains a common and potentially life-threatening AIDS-defining illness [3]. *Histoplasma capsulatum* is a thermally dimorphic fungus, growing in a filamentous mycelial form in the soil and as an encapsulated yeast at 37°C or higher in the body. Acute pulmonary infection occurs via inhalation of dust particles from soil containing *H. capsulatum *spores, or microconidia. At body temperature, microconidia convert to yeasts, which are opsonized with antibody and phagocytosed by alveolar macrophages. Infected macrophages respond by secreting interleukin-12 and activating CD4^+^ T cells [4, 5]. In individuals with intact cellular immunity, activated CD4^+^ T cells subsequently produce interferon-*γ* to promote intracellular killing [4, 5]. However, in individuals with CD4^+^ cell counts <100/mm^3^, macrophages are unable to contain infection. Disseminated infection occurs via lymphohematogenous spread of yeast-filled macrophages into nearby lymph nodes and multiple organs, most commonly the liver, spleen, bone marrow, adrenal glands, and gastrointestinal (GI) tract [2]. While GI involvement is not uncommon in patients with disseminated disease, gastrointestinal histoplasmosis (GIH) rarely comes to clinical attention due to the lack of specific signs and symptoms [6]. We report the unusual case of a 33-year-old patient with advanced AIDS who developed significant upper GI bleeding from fungal lesions in the duodenum leading to clinical presentation with worsening anemia and melanotic stools.

## 2. Case Report

The patient was a 33-year-old Caucasian male with advanced AIDS (CD4^+^ cell count: 59 cells/mm^3^, HIV-1 RNA level of 308,000 copies/mL) and an extensive history of opportunistic infections including prior disseminated histoplasmosis, disseminated *Mycobacterium avium* complex (MAC), *Pneumocystis jirovecii* (PCP) pneumonia, cutaneous varicella-zoster infection, and cytomegalovirus (CMV) gastric ulcer. He had been placed on combination antiretroviral therapy (cART) with emtricitabine/tenofovir and raltegravir as well as opportunistic infection prophylaxis with itraconazole, azithromycin, trimethoprim/sulfamethoxazole (TMP/SMX), and valganciclovir, although the patient reported being poorly adherent to all prescribed medications. He presented to the hospital with a two-week history of worsening cough, shortness of breath, intermittent fever and chills, and black tarry stools. On admission, he appeared cachectic, chronically ill, and in obvious distress. Vital signs were notable for tachycardia and mild tachypnea. Physical examination revealed facial *Molluscum contagiosum*, ulcerations on the right lateral tongue, oropharyngeal thrush, mild diffuse abdominal pain, and moderate hepatosplenomegaly. A complete blood count was significant for pancytopenia with a leukocyte count of 2,800 cells/*μ*L (93% neutrophils, 4% lymphocytes, and 1% monocytes), hematocrit of 20% (down from the patient's baseline of 30%), and platelet count of 28,000/mL. The results of a comprehensive metabolic panel were as follows: sodium 133 mmol/L, potassium 4.1 mmol/L, chloride 102 mmol/L, bicarbonate 22 mmol/L, blood urea nitrogen 16 mg/dL, creatinine 0.76 mg/dL, glucose 103 mg/dL, calcium 7.7 mg/dL, AST 113 units/L, ALT 25 units/L, and alkaline phosphatase 450 units/L.

**Figure 1:**
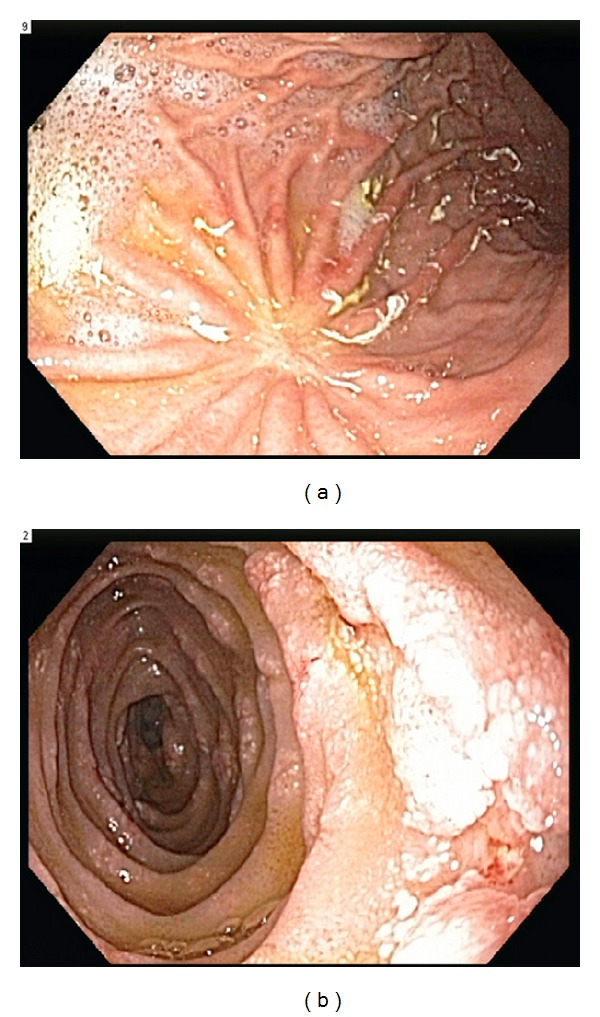
(a) Endoscopic view of the greater curvature of the stomach showing a well-healed scar from prior CMV gastric ulcer. (b) Endoscopic view of the duodenum showing diffuse erosions.

**Figure 2:**
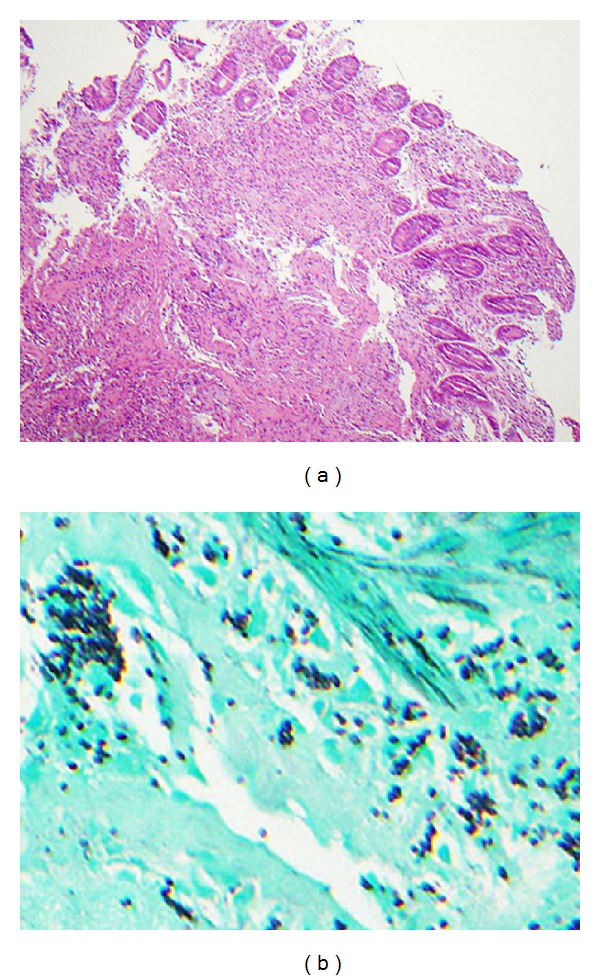
(a) Hematoxylin and eosin stain shows small bowel with granulomatous inflammation. (b) GMS stain highlights intracellular fungal organisms.

On the evening of admission, the patient became febrile to 39.5°C and increasingly tachycardic, tachypneic, and hypoxic requiring supplemental oxygen, several liters of intravenous fluids, and two units of packed red blood cells. A chest radiograph was obtained which showed a diffuse miliary pattern concerning for PCP pneumonia and/or disseminated opportunistic infectious process such as tuberculosis, MAC, or fungal infection. He was started on empiric treatment with vancomycin, piperacillin/tazobactam, high dose TMP/SMX, prednisone, ethambutol, azithromycin, and liposomal amphotericin B. He responded well to broad-spectrum treatment over the next few days and subsequently underwent diagnostic bronchoscopy with bronchoalveolar lavage (BAL). BAL cultures returned positive for *H. capsulatum*. The patient also underwent esophagogastroduodenoscopy (EGD) given his persistent melanotic stools and progressively declining hematocrit (nadir of 18%) requiring multiple blood transfusions. EGD was significant for a well-healed scar on the greater curvature of the stomach from prior CMV gastric ulcer and new diffuse erosions throughout the second, third, and fourth segments of the duodenum (Figure 1). Multiple biopsies were taken which showed duodenal mucosa with granulomatous inflammation. Gomori's methenamine silver (GMS) stain identified macrophages with small intracellular yeasts consistent with disseminated histoplasmosis (Figure 2). Bone marrow involvement was also suspected in light of the patient's significant pancytopenia, but he had previously refused bone marrow biopsy. Urine *Histoplasma* antigen came back markedly elevated at >39 ng/mL (above the upper limit of detection for this particular assay) confirming the diagnosis, and the patient was treated with a two-week course of intravenous liposomal amphotericin B followed by indefinite oral itraconazole.

Following a two-week inpatient hospitalization, the patient demonstrated excellent improvement. All major presenting symptoms were resolved, including the melanotic stools, and his hematocrit was stable at his baseline. By the time of discharge, the patient had regained his prior level of functioning including full ambulatory capacity. Several weeks after discharge, sputum acid-fast culture returned positive for MAC for which he was continued on ethambutol and azithromycin.

## 3. Discussion

In patients with disseminated histoplasmosis, the gastrointestinal (GI) tract is one of the most commonly affected organ systems with approximately 70% of patients demonstrating some GI involvement at autopsy [5, 7]. While gastrointestinal histoplasmosis (GIH) may involve any portion of the GI tract, nearly 90% of lesions involve the lower GI tract, most commonly the ileocecal region or colon [8]. This is thought to be due to the abundance of gut-associated lymphoid tissue (GALT) in these areas, such as Peyer's patches in the terminal ileum, which may serve as entry sites for macrophages filled with *H. capsulatum *yeasts [9]. Histologically, GIH first appears as focal lesions in the submucosa and lamina propria, but the gross appearance of these lesions is highly variable [5, 10]. On endoscopy, GI lesions may appear as segments of inflamed or thickened bowel, ulcerations, strictures, polyps, or tumor-like lesions [8, 10]. This variation in gross appearance makes GIH a great imitator of other GI disorders, most notably inflammatory bowel diseases and GI malignancies [11–13].

Although common at autopsy, GIH is rarely recognized clinically with GI symptoms being reported in <10% of cases of disseminated infection [6, 7]. This may be due, in part, to the nonspecific manifestations of GIH, namely, fever, weight loss, diarrhea, and abdominal pain [8]. In a minority of cases, however, GIH may present with more specific manifestations such as dysphagia or odynophagia with upper GI tract involvement and bowel obstruction, bowel perforation, or hematochezia with lower GI tract involvement [14–17]. Our case represents a unique presentation of GIH given both an atypical location (upper GI tract) and unusual symptomatology (melena).

In the largest case series of GIH in AIDS patients, duodenal disease was appreciated in <4% of cases, making it one of the rarest sites of GI involvement [8]. Of the few case reports of duodenal histoplasmosis described in the literature, most have presented with nonspecific symptoms, such as weight loss or abdominal pain, or no symptoms at all [18, 19]. To our knowledge, this is the first case report of duodenal histoplasmosis leading to clinically significant bleeding, manifesting with melanotic stools and worsening anemia. The presence of melena implies either significant bleeding of lesions in the upper GI tract (i.e., proximal to the ligament of Treitz) or slow bleeding of lesions in the lower GI tract. Most cases of melena secondary to GIH can be attributed to the latter etiology given its strong predilection for the lower GI tract [11]. However, it should be noted that the majority of bleeding lesions in the lower GI tract present with hematochezia, not melena. One rare case of true upper GI bleeding and resultant melanotic stools involved GIH with significant esophageal involvement and diffuse ulcerations [20].

Given the discrepancy between autopsy data and clinically reported data, we suspect that the incidence of GIH is underestimated and clinicians should consider this diagnosis in AIDS patients at risk, specifically those with advanced immunosuppression (i.e., CD4^+^ cell counts <100 cells/mm^3^) who reside in endemic areas (Ohio or Mississippi river valleys as well as temperate regions of Central and South America, Africa, and Asia) and/or have a prior history of histoplasmosis. While the clinical presentation of GIH is often vague and nonspecific, it should be considered on the differential diagnosis in patients with a variety of different upper or lower GI symptoms. In light of our case report, we argue that melena, although less common than hematochezia, should be recognized as a possible initial manifestation of GIH and should prompt further diagnostic investigation. As an initial noninvasive test, we recommend checking a urine histoplasma quantitative antigen EIA. Patients with a positive test may then be subject to a further diagnostic workup, including upper and/or lower endoscopy with biopsy and GMS stain for intracellular yeasts. Patients with GIH are readily treatable with a two-week course of intravenous liposomal amphotericin B followed by long-term treatment with itraconazole, often indefinitely in patients with chronic immunosuppression [21, 22]. We maintain that early recognition and treatment of GIH can help to improve patient outcomes and prevent disease recurrence.
